# Salvage Perioperative Interstitial High-Dose-Rate Interventional Radiotherapy (Brachytherapy) for Local Recurrences of the Chest Wall Following Mastectomy and Previous External Irradiation

**DOI:** 10.3390/cancers15030614

**Published:** 2023-01-18

**Authors:** Tamer Soror, Maggie Banys-Paluchowski, Corinna Melchert, Dirk Rades, Achim Rody, Kerstin Muras, Meiting Xie, György Kovács

**Affiliations:** 1 Radiation Oncology Department, University of Lübeck/UKSH-CL, 23562 Lübeck, Germany; 2 Radiation Oncology Department, National Cancer Institute (NCI), Cairo University, Cairo 11796, Egypt; 3 Department of Gynecology/Breast Unit, University Hospital Schleswig-Holstein Campus Lübeck, 23538 Lübeck, Germany; 4 Gemelli-INTERACTS, Università Cattolica del Sacro Cuore, 00168 Rome, Italy

**Keywords:** breast cancer, chest wall recurrence following mastectomy and irradiation, perioperative interventional radiotherapy (brachytherapy)

## Abstract

**Simple Summary:**

Breast cancer patients who were treated with mastectomy and postoperative external irradiation may suffer from tumor relapse in the chest wall. The local treatment of such conditions prevents complications such as ulceration, bleeding, infection, offensive odor, and major psychological stress. In this study, we investigated an approach combining both surgical removal of the tumor and implanting plastic tubes for postoperative interventional radiotherapy in the operated regions. After accurate and personalized computer-based 3-dimensional planning, a small computer remoted radioactive source is introduced through the tubes to re-irradiate the operated region. The procedure led to local control of the tumor in 82% of the patients in five years. A few patients (8.9%) suffered from severe but manageable complications during the follow-up period. We believe that this perioperative and interdisciplinary treatment may be a valid option to help locally recurrent and previously irradiated breast cancer patients as it offers good oncological results and a low rate of severe complications.

**Abstract:**

(1) Background: To investigate the technical feasibility, safety, and efficacy of interstitial perioperative high-dose-rate interventional radiotherapy (HDR-IRT, brachytherapy) as a local salvage treatment combined with surgery for local chest wall recurrences following mastectomy and subsequent external beam radiation treatment (EBRT). (2) Methods: A retrospective analysis of 56 patients treated with interstitial HDR-IRT in combination with local surgery of a chest wall recurrence of breast cancer after previous treatment with mastectomy and EBRT from 2008 to 2020. (3) Results: Local recurrence following HDR-IRT was encountered in seven (12.5%) patients. The 1-year local recurrence-free survival (RFS), 3-year RFS, and 5-year RFS were 91%, 82%, and 82%, respectively. The 1-year overall survival (OS), 3-year OS, and 5-year OS was 85.5%, 58%, and 30%, respectively. Acute grade 1–2 radiation dermatitis was observed in 22 (39.3%) patients. Late ≥grade 3 toxicities were encountered in five (8.9%) patients. (4) Conclusions: Salvage perioperative interstitial high-dose-rate interventional radiotherapy (brachytherapy) combined with surgery seems to be an effective interdisciplinary management with acceptable treatment-related toxicity for local recurrences of the chest wall following mastectomy and previous external irradiation.

## 1. Introduction

Local and locoregional recurrences following mastectomy and external irradiation in locally advanced breast cancer patients remain a frequent clinical problem. The rate of local recurrence (LR) in the ten years after mastectomy and post-operative irradiation may be as low as 1.6% in node-negative disease or as high as 8.1% in node-positive breast cancer [1,2,3]. Without post-operative irradiation, the rate of LR may reach 26% [3]. Local recurrences may be accompanied by other nodal or distant metastases in about one-third of the patients [1]. Whether isolated or as a part of a metastatic disease, uncontrolled LR could present with pain, ulceration, bleeding, infection, offensive odor, and major psychological stress. Patients with uncontrolled LR will consequently suffer from impaired quality of life; 62% of such patients reported a severe impairment of all quality of life indices [1]. The chest wall is the most common site of locoregional recurrence, whether as an isolated recurrence or accompanied by regional nodal recurrence or by distant metastases [4].

Curative local treatment options are usually limited. Surgery for resectable LR may be a suitable option with the challenge of local tissue reconstruction. In locally advanced lesions, there is a significant risk of positive margins or even gross residual [1,2]. On the other hand, reirradiation with external beam radiation treatment (EBRT) carries a potential high risk of radiation toxicities such as brachial plexopathy, soft tissue or bone necrosis, lymphedema of the arm, severe chest wall fibrosis, and lung fibrosis [5,6,7]. Reirradiation with hyperthermia had acceptable results with 14% of late grade 3 or higher toxicities [8,9]. Using contact brachytherapy with surface molds in extreme hypofractionation, in a split-course technique, or in combination with hyperthermia has been investigated [10,11,12]. Unfortunately, contact brachytherapy with surface molds is mainly limited to skin metastases as the dose is usually prescribed to a maximum depth of 5 mm [10,11,12]. 

A combined approach of curative or debulking surgery with perioperative interstitial high-dose-rate interventional radiotherapy (HDR-IRT, brachytherapy) was rarely investigated [13,14]. HDR-IRT is a highly accurate and conformal technique of irradiation. The sharp dose gradient ensures sparing of the adjacent tissues and organs at risk (OAR). Therefore, HDR-IRT is considered a suitable salvage option for the reirradiation of locally recurrent tumors following previous EBRT [15,16,17].

This retrospective analysis investigates the technical feasibility, safety, and efficacy of perioperative HDR-IRT as a salvage local treatment combined with surgery for resectable local recurrences of the chest wall following mastectomy subsequent to EBRT. To our knowledge, this is by far the largest published cohort of patients for such a treatment approach.

## 2. Patients and Methods

### 2.1. Patients

We reviewed all medical records of patients who received salvage HDR-IRT from January 2008 to December 2020. Only patients who had a history of mastectomy and previous EBRT were included in the analysis. A multidisciplinary tumor board (MDTB) discussed the treatment decision for each patient individually. Eligible patients were restaged through a physical examination, laboratory investigations, radiographic and/or isotopic scans before treatment, and provided signed informed consent before treatment. All patients received complementary systematic treatments according to national guidelines based on the MDTB decision.

### 2.2. Interventional Radiotherapy (Brachytherapy)

**Figure 1:**
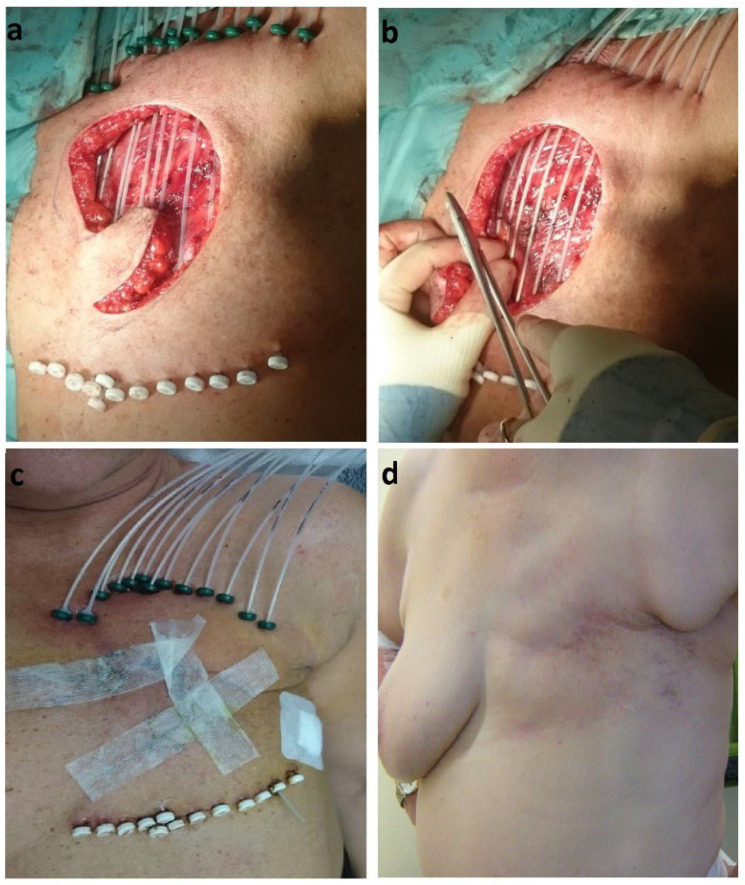
(**a**) and (**b**): intraoperative catheter implantation and suturing; (**c**): during the irradiation; (**d**): 5 years after high-dose-rate interventional radiotherapy.

At the time of the surgical resection of the LR, the interventional radiotherapy expert evaluated the anatomy situation and potential micro- or macroscopic residual tumor locations together with the surgeon. The macroscopic tumor bed was marked with metallic surgical clips. Interstitial plastic tubes were implanted and sutured to the tumor bed in single plane (monoplane implant) with minimum two-centimeter distance to the surgical margin on the skin. The tubes were kept parallel to each other with an interspacing distance of 8–12 mm over the target area. In case of needed surgical reconstruction, interstitial tubes were implanted afterwards but before final suture (Figure 1). 

**Figure 2:**
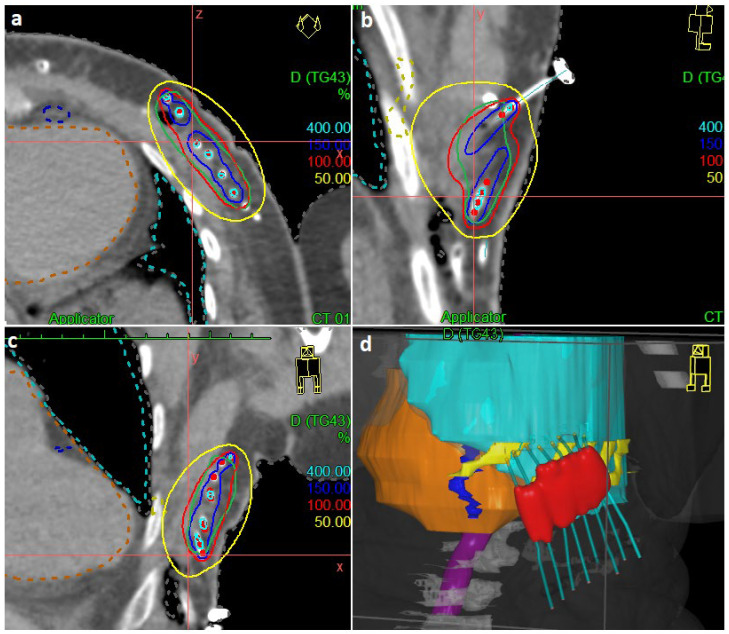
Representative samples of the dose distribution: (**a**): transversa; (**b**): sagittal; (**c**): coronal; (**d**): three-dimensional reconstruction. Green: clinical target volume; yellow: rips; orange: heart; red: reference isodose line.

Thin-slice computed tomography (CT) was performed on the second or the third postoperative day and digitally forwarded into the treatment planning system (TPS). The three-dimensional (3D) reconstruction of the CT images was used for tubes reconstruction, volume definition, and dose planning/optimization on the TPS (Oncentra planning; Elekta Brachytherapy, Veenendaal, Netherlands). The clinical target volume (CTV) included the estimated tumor bed, the surgical clips, and a safety margin of 10–20 mm according to the size of the reported microscopic surgical margin and excluding the skin. A minimum distance (3–5 mm) was kept between the reference isodose line and the skin surface, as well as a maximum 10 mm distance between the lateral tube and the reference isodose line [14]. The radiation was started according to the type of surgery and condition of the patient usually 4–5 days after surgery and the prescribed dose was delivered in twice-daily consecutive fractions with a minimum 6 h interval. Figure 2 shows a sample of the 3D dose distribution.

### 2.3. Follow-up

Patients were regularly examined every 3 months for 3 years, then every 6 months for 2 years, then annually. Treatment-related toxicities were graded according to the common terminology criteria for adverse events 5.0.

### 2.4. Statistical Analysis

Results were reported as an absolute value, median with range, or as mean with standard deviation. Overall survival (OS) was defined from the end of HDR-IRT to the date of the last follow-up visit or to the date of death. Recurrence-free survival (RFS) was defined from the end of HDR-IRT to the date of the last follow-up visit or to the date of confirming a local recurrence. Probability estimates of RFS and OS were calculated using Kaplan–Meier analysis. Multivariable logistic regression analysis was performed in order to identify factors independently associated with local recurrence; patients with missing data were excluded from this analysis. Examined factors included age, T stage, initial lymph node status, histologic type, histologic grade, ER/PR status, Her2 status, surgical margin status, hormonal treatment, and chemotherapy treatment. Statistical analyses were performed using SPSS-V.20 (IBM-Corp. Armonk, NY, USA).

## 3. Results

### 3.1. Patient and Tumor Characteristics

We identified 56 patients who received salvage HDR-IRT for chest wall recurrences following mastectomy and previous external irradiation. Table 1 summarizes the patients and disease characteristics. The median initial age of patients at the time of diagnosis with breast cancer was 55 years (range: 31–72). Mastectomy was performed as the primary surgical operation in 43 (75.4%) patients, while in 14 (24.6%) patients, mastectomy was performed for LR in the breast. All patients had received previous EBRT as part of the primary treatment. The median dose of EBRT was 50.4 Gy (range: 40–60.4). The median interval between EBRT and HDR-IRT was 12.5 months (range: 7–288).

The median age of the patients at HDR-IRT was 70 years (range: 37–89). After local resection of the recurrence, five patients received an abdominal rotational flap to reconstruct the surgical defect. Latissimus dorsi myocutaneous flap was used in one patient. The LR was macroscopically resected in all patients. Five (8.9%) patients had microscopically positive margins. 

### 3.2. HDR-IRT Treatment Characteristics

The median number of catheters inserted during the procedure was 6 (range: 4–16). The median clinical target volume (CTV) was 25.5 cc (range: 16–46). The median dose was 30 Gy (range: 25–36). Other dosimetry parameters are summarized in Table 2.

### 3.3. Treatment Outcome and Related Toxicities

**Figure 3:**
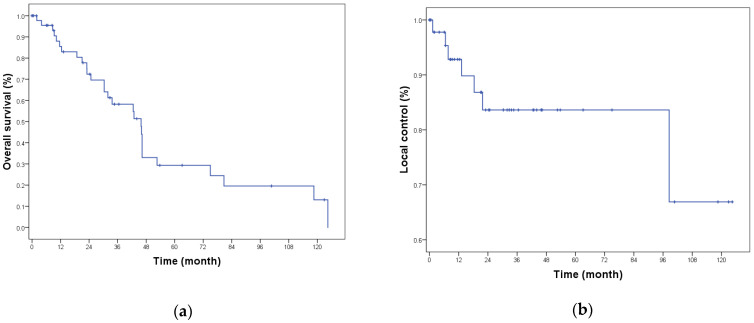
(**a**) Overall survival; (**b**) local recurrence-free survival.

The median follow-up time was 30.3 months (range: 0.9–124.4). Local recurrence following HDR-IRT was encountered in seven (12.5%) patients; in those seven patients, the median time to develop LR was 13.2 months (range: 1.3–89.3) following HDR-IRT. Only two of them (2/7) had positive surgical margin. The one-year (1-y) local recurrence-free survival (RFS), 3-y RFS, and 5-y RFS were 91%, 82%, and 82%, respectively (Figure 3). Multivariable analysis did not identify any risk factor predictive for local recurrence.

During follow-up, 28 (50%) patients died. Among dead patients, five patients had local recurrence (17.9%). Death resulted from progressive metastatic disease in 26 patients and unknown causes in 2 patients. The mean time of death for those two patients was 66.4 months after the HDR-IRT. Among living patients at the time of analysis, six patients had evidence of disease. Four of them had distant metastases with or without locoregional recurrence and two patients had only regional nodal recurrence. The one-year (1-y) overall survival (OS), 3-y OS, and 5-y OS were 85.5%, 58%, and 30%, respectively (Figure 3).

Acute radiation dermatitis grade 1 and 2 was observed in 22 (39.3%) patients. The recorded treatment-related late toxicities were mostly grade 1–2. Late skin toxicities (grade 1–2) were documented in 19 (33.9%) patients. Fourteen patients had grade 1–2 fibrosis, and three patients had fibrosis grade 3 in the HDR-IRT field. One patient had chronic wound complications and needed a skin graft. One patient developed localized soft-tissue necrosis along the pathway of one of the HDR-IRT catheters, which was surgically treated. The total number of observed late ≥ grade 3 toxicities was five (8.9%) (Table 3). Other late toxicities that were investigated and not observed included pathological rib fracture, radiation-induced lung disease, and radiation-induced heart disease. 

## 4. Discussion

Although most patients with local recurrence will eventually suffer from distant metastases [1,2], aggressive local treatment is required to prevent local complications and deterioration in the quality of life [1,4]. Local treatment options following mastectomy and external irradiation are limited. Surgery is the primary local treatment option in resectable lesions. The rate of local recurrence following surgery is 20% [18], and the 5-year local recurrence-free rate is 51% [19]. In a multi-institutional review of repeated irradiation of the chest wall or the breast for recurrent breast cancer, the overall complete response rate was 57% among 81 patients with only 12 months median follow-up time [20]. In a recent study by Fattahi et al., reirradiation using photon +/− electrons or protons for chest wall recurrences to a median dose of 45 Gy resulted in 74.6% 2-year locoregional recurrence-free survival. The cohort included 72 patients who were curatively (38%) or postoperatively (62%) re-irradiated. After a median follow-up of 22 months, the rate of severe late toxicity (≥grade 3) was 13% [5]. 

In a large study by Oldenborg et al., hyperthermia was applied in addition to EBRT in order to sensitize tumor cells among 414 patients with irresectable locoregional recurrent breast cancers. The overall clinical response rate was 86%, and the rate of severe late toxicity (≥grade 3) was 24% [21]. In another study that included 169 patients treated with reirradiation plus hyperthermia for recurrent breast cancer en cuirasse, the overall clinical response rate was 72%. Late ≥grade 3 toxicity was recorded in 14% of patients [9].

Pulsed reduced dose rate (PRDR) EBRT delivers the radiation dose at a significantly lower dose rate, which is believed to reduce toxicity to normal tissue through increasing the sublethal damage repair of normal tissues [22]. Burr et al. analyzed the results of PRDR-EBRT for reirradiation of recurrent breast cancer. The 2-year locoregional recurrence-free survival was 73.8%. Among the 43 patients who were included in the analysis, 18.6% had grade 3 late toxicity [23]. 

Furthermore, the use of proton reirradiation for locally or locoregionally recurrent breast cancer was investigated in few reports with a small number of patients [5,24,25]. Furthermore, the studies of proton reirradiation included chest wall recurrences as well as breast or lymph node(s) recurrences. Therefore, solid conclusions regarding its use in the treatment of isolated chest wall recurrences could not be obtained [5,24,25].

Contact brachytherapy with surface molds with or without hyperthermia is predominantly used to treat skin metastases. Besson et al. evaluated 43 patients who were treated with 4–6 fractions of HDR-brachytherapy twice weekly to 24 Gy mean total dose. The 3-year in-field local control was 51% with 11.6% severe late toxicity [10]. Harms et al. treated 58 patients with pulsed-dose-rate (PDR) contact brachytherapy. All patients were treated with a split course of two fractions of 20 Gy. The 3-year local recurrence-free survival was 75%. The rate of severe late toxicity was 67% [12]. Auoragh et al. investigated the addition of hyperthermia to contact brachytherapy in 18 patients. The 5-year local recurrence-free survival was 56%, and 17% of the patients had severe late complications.

In a study by Niehoff et al., 32 patients were treated with both surgical resection and HDR or PDR interstitial brachytherapy. The PDR-brachytherapy mean dose was 30 Gy, and the HDR-brachytherapy mean dose was 28 Gy in twice-daily fractions. The mean follow-up time was 13 months. Local control was achieved in 20/32 patients. Only one patient experienced grade 3 late toxicity [14].

In the present study, we reported the results of salvage perioperative interstitial HDR-IRT for chest wall recurrences following mastectomy and previous external irradiation. Multimodal management that includes surgery for resectable lesions, radiation +/− hyperthermia, and possible systemic treatment may achieve a high rate of local control and could be curative in a subset of patients with an isolated chest wall recurrence [26]. In the current analysis, all treated lesions were macroscopically resected, and the HDR-IRT catheters were immediately intraoperatively implanted, which ensured an ideal estimation of the target volume. This combined approach aimed at maximum local control. The median dose of HDR-IRT was 30 Gy delivered in twice-daily fractions, which is a similar treatment approach to that reported by Niehoff et al. 

In contact brachytherapy, skin and subcutaneous tissue receive significantly higher doses than the prescribed dose as the dose is typically prescribed to a depth of 5 to 7 mm. We used CT-based planning and optimization, and the skin was excluded from the reference dose. Additionally, a maximum 10 mm distance was allowed between the lateral tube and the reference isodose line in order to avoid very high dose regions within the target. This could explain the low rate of late ≥grade 3 toxicities (5/56, 8.9%) among our patients compared to other radiation techniques used for reirradiation, including contact brachytherapy. The sharp dose gradient of HDR-IRT resulted in a low mean dose to the ipsilateral lung (3.2 Gy). Likewise, the mean dose to the heart in left-sided tumors was significantly low (2.8 Gy). Among our patients, there was no observed radiation-induced heart or lung disease. Studies that used different EBRT techniques for reirradiation did not discuss the incidence of such complications.

The approach of combining local surgery with interstitial HDR-IRT as a salvage option for tumor recurrences after previous irradiation has been investigated in different tumor entities [14,15,26,27,28,29]. In a large matched cohort analysis of the GEC-ESTRO, combining both local surgery and interstitial HDR-IRT as a salvage management for ipsilateral recurrence of breast cancer following conservative breast therapy resulted in similar oncological outcomes compared to salvage mastectomy [30]. In the present study, the combined approach of macroscopic resection of the LR followed by HDR-IRT resulted in 82% 5-year local recurrence-free survival, which is higher than the local control rates reported by other studies.

## 5. Conclusions

Salvage perioperative interstitial high-dose-rate interventional radiotherapy (brachytherapy) combined with surgery seems to be a feasible and effective interdisciplinary management for local recurrences of the chest wall following mastectomy and previous external irradiation. Intraoperative target definition and clip documentation, as well as CT-based personalized dose optimization and dose painting using the stepping-source technology, offer good target coverage and spares organs at risk, which consequently leads to good local control rates and acceptable treatment-related toxicity. 

## Figures and Tables

**Table 1:** Patients and disease characteristics.

Characteristic	Value	Number (%)
Initial T stage	T0 T1 T2 T3 T4 Unknown	1 (1.8%) 11 (19.6%) 22 (39.3%) 4 (7.1%) 6 (10.7%) 12 (21.4%)
T stage at salvage treatment	T1 T2 T3 T4	19 (33.9%) 32 (57.1%) 3 (5.4%) 2 (3.6%)
Initial lymph node status	Negative Positive Unknown	20 (35.7%) 24 (42.9%) 12 (21.4%)
Histology type	Ductal carcinoma in situ Non-special type Lobular carcinoma Other	1 (1.8%) 39 (69.6%) 9 (16.1%) 7 (12.5%)
Initial histologic grade	Grade 1 Grade 2 Grade 3 Unknown	3 (5.4%) 21 (37.5%) 17 (30.4%) 15 (26.7%)
Histologic grade at salvage treatment	Grade 1 Grade 2 Grade 3	3 (5.4%) 27 (48.2%) 26 (46.4%)
Initial surgical margin status	Negative Positive	56 (100%) 0 (0%)
Surgical margin status at salvage treatment	Negative Positive	51 (47.1%) 5 (8.9)
Estrogen/progesterone receptor status	Positive Negative Unknown	39 (69.6%) 12 (21.4%) 5 (8.9%)
HER2-status	Positive Negative Unknown	22 (39.3%) 28 (50%) 6 (10.7%)
Primary surgical operation	Mastectomy Breast conserving surgery	43 (75.4%) 14 (24.6%)
Previous hormonal treatment		39 (69.6%)
Previous chemotherapy		43 (76.8%)

**Table 2:** Dosimetry parameters of the HDR-IRT.

Parameter	Median (Range)
HDR-IRT dose Total dose Dose per fraction Number of fractions	30 Gy (25–36) 2.5 Gy (2.5–4) 10 (8–12)
Clinical target volume (CTV)	
Volume D90% V100% V150%	25.5 cc (16–46) 93.8% (89.5–102) 87.2% (84.3–92.8) 37.2% (33.1–42.2)
Ipsilateral lung mean dose	3.2% (2.7–3.8)
Heart mean dose (in left-sided tumors)	2.8% (2.2–3.3)

HDR-IRT: high-dose-rate interventional radiotherapy; Gy: Gray; cc: cubic centimeter; D90%: the dose in percentage that covers 90% of CTV; V100: the volume in percentage that is receives 100% of the prescribed dose; V150%: the volume in percentage that receives 150% of the prescribed dose.

**Table 3:** Treatment-related toxicities.

Encountered Toxicity	Number (%)	Severity
Acute radiation dermatitis	22 (39.3%)	Grade 1–2
Late skin toxicities	19 (33.9%)	Grade 1–2
Fibrosis	14 (25%) 3 (5.4%)	Grade 1–2 Grade ≥3
Chronic wound complications	1 (1.8%)	Grade ≥3
Necrosis	1 (1.8%)	Grade ≥3

## Data Availability

The data presented in this study are available in this article.
